# Coinfection of gastrointestinal parasites with paratuberculosis in naturally infected sheep

**DOI:** 10.14202/vetworld.2024.2577-2585

**Published:** 2024-11-22

**Authors:** Rami M. Mukbel, Wael M. Hananeh, Asya Radhi, Zuhair Bani Ismail

**Affiliations:** 1 Department of Basic Veterinary Medical Sciences, Faculty of Veterinary Medicine, Jordan University of Science and Technology, Irbid, Jordan; 2 Department of Veterinary Pathology and Public Health, Faculty of Veterinary Medicine, Jordan University of Science and Technology, Irbid, Jordan; 3 Department of Clinical Veterinary Medical Sciences, Faculty of Veterinary Medicine, Jordan University of Science and Technology, Irbid, Jordan

**Keywords:** coinfection, *Mycobacterium avium paratuberculosis*, parasite-pathogen-host relationship, sheep

## Abstract

**Background and Aim:**

*Mycobacterium avium* subspecies paratuberculosis (MAP) causes Johne’s disease (paratuberculosis), which is a chronic wasting disease. No previous study has been found to investigate the coinfection of gastrointestinal parasites with paratuberculosis. This study aimed to investigate the relationship between paratuberculosis and gastrointestinal parasitism in naturally infected local Awassi (AS) and imported Tsurcana (TS) sheep.

**Materials and Methods:**

A total of 207 sheep (118 AS and 89 TS) were randomly selected from local butcher shops and slaughterhouses. Fecal and tissue samples from the ileum and mesenteric lymph nodes were collected. Fecal samples were screened for the presence of parasitic ova and oocysts. Hematoxylin-and-eosin-stained and Ziehl–Neelsen-stained tissues were examined for evidence of inflammation, acid-fast bacteria, and parasitic structures. Risk factor analysis was performed using multivariate logistic regression analysis.

**Results:**

Mucosal thickening and corrugation of the ileum were found in AS sheep (74/113 [65.5%], 10/113 [8.8%], respectively) and TS sheep (21/88 [23.9%], 8/88 [9.1%], respectively). Histopathologically, diffuse multibacillary/histiocytic form was detected only in the AS sheep breed. AS sheep had higher levels of acid–fast bacteria in the ileum (70/113 [61.9%]) compared with TS sheep (34/88 [38.6%]). In addition, parasitic structure and severe eosinophilic inflammation were detected in AS (10/113 [8.8%], 108/113 [95.6%], respectively) and TS sheep (2/88 [2.3%], 70/88 [79.5%], respectively). Parasitic infections were detected in fecal samples from 15/118 (12.7%) AS sheep and 11/89 (12.4%) TS sheep. Coinfection between gastrointestinal parasites and paratuberculosis was detected histologically in 8/118 (7.1%) and 2/88 (2.3%) AS and TS sheep, respectively.

**Conclusion:**

Risk factor analysis indicated that the ileum from the AS sheep breed was 2.164 times more likely to have acid–fast bacteria and 2.874 times more likely to have eosinophilic infiltrations than the TS sheep breed. Results of this preliminary study may indicate the role of the sheep in the pathogenesis and shedding of MAP.

## Introduction

Johne’s disease (paratuberculosis) is a chronic, wasting, and incurable infectious ruminant disease [1]. The disease is caused by an infection with *Mycobacterium avium* subspecies *paratuberculosis* (MAP), resulting in chronic granulomatous enteritis characterized by chronic diarrhea and weight loss [1]. In the intestine, bacteria reside in the macrophages of the intestinal lymphoid tissue, lamina propria, and nearby mesenteric lymph nodes [2]. Paratuberculosis is a widespread disease that causes massive financial losses for livestock industries [1]. Severe economic losses result from reduced milk production, loss of body condition despite normal appetite, early culling, and mortality [3]. The disease is characterized by a long incubation period, which makes it challenging to diagnose and control [4]. Moreover, there are no curative therapies for paratuberculosis, and its control relies on the implementation of strict biosecurity measures to reduce the spread of MAP [5].

In field conditions, animals are exposed to nume-rous pathogens [6]. Although a previous study by Venter *et al*. [7] has focused on a single infectious agent, co- infection with multiple pathogens within the same host likely occur in sick animals. Mabbott [8] has suggested that co-infecting pathogens interact with and modulate the host’s immune response to influence disease transmission, pathogenesis, and progression. For example, a protective effect has been found between coinfection with *Trichuris suis* and Crohn’s disease in humans [9].

Given the similarities between Crohn’s disease and Johne’s disease, gastrointestinal parasites in ruminants may modulate the host’s immune response and alter the pathology and immunology of Johne’s disease. No previous study has investigated the association between paratuberculosis and other diseases, particularly gastrointestinal parasitism in sheep. This study aimed to investigate the relationship between paratuberculosis and gastrointestinal parasitism in naturally infected local Awassi (AS) and imported Tsurcana (TS) sheep

## Materials and Methods

### Ethical approval

This study was reviewed and approved by the Animal Care and Use Committee of Jordan University (Approval No. 02/2020). The samples were collected from the slaughterhouses and butcher shops.

### Study period and location

The study was conducted from December 2019 to August 2021. The samples were collected from local butcher shops in the Irbid, Ramtha, and Al-Aghwar regions, Jordan University of Science and Technology Animal Health Center, Amman City slaughterhouse, and Mafraq City slaughterhouse.

### Animals

Two different breeds of sheep were used in this study: AS and TS sheep from Romania. The total number of sheep included in the study was 207 (118 AS and 89 TS). All samples were obtained from animals older than 1.5 years.

### Gross examination and sample collection

Tissue (ileum and associated mesenteric lymph nodes) and fecal samples were collected from all selected sheep during slaughter. The number of samples collected was as follows: Ileum (201), associated lymph nodes (192), and fecal samples (207). Before sample collection, the carcass was carefully inspected, and any abnormalities involving any body organ were recorded. Samples were excluded from animals with abnormalities involving organs other than the gastrointestinal system. The ileum was grossly examined for mucosal thickness and intestinal wall corrugation. In addition, the ileal-associated mesenteric lymph nodes were examined for any significant gross lesions, and gross pathological findings were recorded. Samples were only collected if the ileum, associated lymph nodes, or both tissues showed gross lesions suggestive of Johne’s disease. Tissue samples for histopathological examination were placed in jars containing 10% buffered formalin. Fecal samples were collected from each animal rectally using clean, dry examination gloves and placed in clean storage containers on ice packs. Tissue and fecal samples were transported to the laboratory for processing within 2–4 h.

### Histopathological examination

In the laboratory, representative tissue samples were collected from the ileum and associated mesenteric lymph nodes and were fixed in 10% buffered formalin for at least 24 h. The fixed tissues were cut and processed routinely in an automatic tissue processor. Paraffin-embedded tissue blocks were prepared, and 3–5 μm thick tissue sections were prepared and mounted on glass slides. Sections from each tissue sample were stained with two different stains, hematoxylin and eosin (H&E) and Ziehl–Neelsen (ZN), in accordance with previously published methods [10].

Stained tissue sections were examined undera light microscope (Motic, China). In accordance with previously published methods, lesions involving the ileum were classified into four different forms of the disease: focal, multifocal, diffuse multibacillary/histiocytic, and diffuse paucibacillary/lymphocytic (Table-1) [11, 12]. Tissue sections from the mesenteric lymph nodes were examined for epithelioid cells, giant cells, and mineralization.

**Table-1:** Classification of Johne’s disease histopathological lesions in the ileum [11, 12].

Focal form	Presence of small well-demarcated granulomas in the lymphatic tissue of the intestine and associated lymph nodes.
Multifocal forms	Presence of small granulomas in lymphoid tissue and in the lamina propria. However, the normal histological structure was not drastically changed.
Diffuse multibacillary/histiocytic form	Presence of sheets of epithelioid macrophages with numerous intracytoplasmic acid–fast bacteria.
Diffuse paucibacillary/lymphocytic form	The presence of numerous lymphocytes within the lamina propria surrounding scattered granulomas with macrophages containing few or no acid–fast bacteria.

Tissue sections were also examined for parasitic structures and eosinophilic infiltration. Parasitic structures were classified as protozoa (*Coccidia* and *Eimeria* spp.), nematodes (*Strongyle* or *Nematodirus* spp.), or mixed infection with protozoa and nematodes. Eosinophilic infiltration was classified as mild (fewer than five eosinophils per field), moderate (5–20 eosinophils per field), and severe (more than 20 eosinophils per field).

### Fecal parasite examination

The fecal floatation technique was conducted to examine the fecal samples for parasitic ova in accordance with a previously published method [13]. Fecal floatation results were classified into protozoa (coccidian oocysts), nematode ova (*Strongyle* and *Nematodirus* spp.), and mixed infections, in which protozoa and nematode ova were detected.

### Statistical analysis

The statistical analysis was performed using the IBM statistical software (version 26; IBM SPSS Statistics for Macintosh, IBM Corp. NY, USA). Potential risk factors with p ≤ 0.02 (χ^2^; two-sided test) and no collinearity (r ≤ 0.6) were considered when building the final multivariate logistic regression model using manual logistic regression analysis. Only variables with p ≤ 0.05 were considered statistically significant and were included in the final model. The final model was subjected to the Hosmer and Lemeshow goodness-of-fit test.

## Results

### Animals

The ages of the animals ranged from 1.5 to 5 years. The distribution of animals according to age was 1.5–2 years (60%) and 3–5 years (40%). The age ratio of AS sheep was 1.5–2 years (30%) and 3–5 years (70%). All TS sheep involved in the study were 1.5–2 years old.

### Gross examination

Gross lesions detected in the ileum comprised mucosal thickening in 95/201 (47.3%) and mucosal corrugation in 18/201 (9.0%) of all sheep of both breeds. In imported TS sheep, 21/88 (23.9%) had mucosal thickening, and 8/88 (9.1%) had mucosal corrugation in the ileum. In local AS sheep, 74/113 (65.5%) had mucosal thickening, and 10/113 (8.8%) had mucosal corrugation in the ileum.

Gross lesions involving the mesenteric lymph nodes included enlargement of the nodes in 103/192 (53.6%) and mineralization and enlargement in 9/192 (4.7%) samples. Lymph node enlargement was detected in 52/78 (66.7%) and 51/114 (44.7%) TS and AS sheep, respectively, while mineralization and enlargement were only recorded in AS sheep at 9/114 (7.9%).

### Histopathological examination of the ileum and mesenteric lymph nodes

Out of 201 samples of ileum from both breeds, 128/201 (63.7%) had histopathological lesions consistent with Johne’s disease (Table-2). The intestinal villi were severely distended with variable degrees of mucosal thickening. Heterogeneous populations of inflammatory cells (primarily mononuclear cells) infiltrated the mucosa and submucosa. Lymphangitis was also observed and characterized by the infiltration of lymphocytes and macrophages. There was submucosal edema and dilated lymphatics.

**Table-2:** Results of the histopathological examination of the ileum.

Parameters	Number (%)		

	All sheep	Tsurcana sheep	Awassi sheep
Histological features of ileal lesions			
Normal	73 (36.3)	41 (46.6)	32 (28.3)
Focal form	36 (17.9)	22 (25.0)	14 (12.4)
Multifocal forms	34 (16.9)	12 (13.6)	22 (19.5)
Diffuse multibacillary/histiocytic form	18 (9.0)	0 (0)	18 (15.9)
Diffuse paucibacillary/lymphocytic form	40 (19.9)	13 (14.8)	27 (23.9)
Eosinophilic inflammation			
Absent	23 (11.4)	18 (20.5)	5 (4.4)
Mild	50 (24.9)	26 (29.5)	24 (21.2)
Moderate	48 (23.9)	14 (15.9)	34 (30.1)
Severe	80 (39.8)	30 (34.1)	50 (44.2)

**Figure-1:**
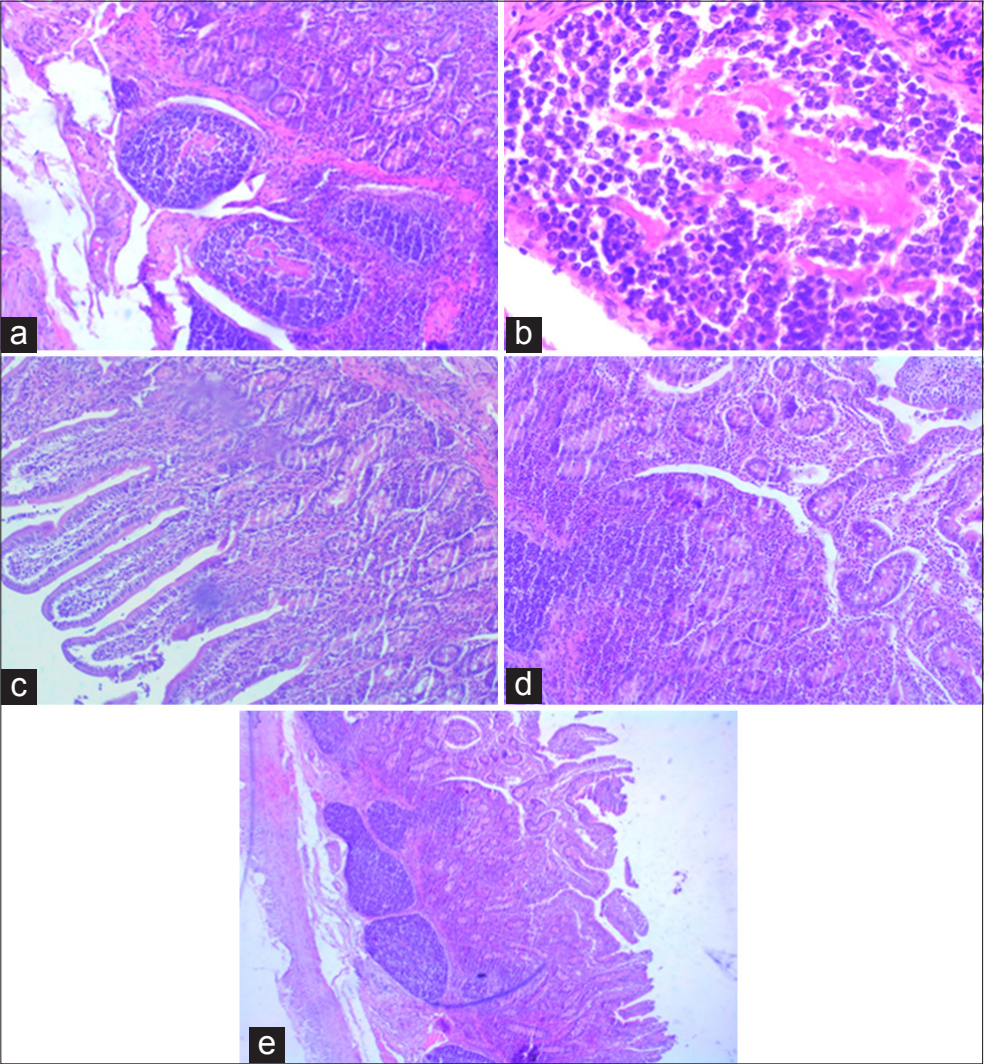
Histopathological classification of intestinal lesions in the ileum caused by *Mycobacterium avium* subspecies *paratuberculosis* in sheep. (a and b) Focal form: small granuloma formed by macrophages located in the lymphoid tissue of the ileum (Peyer’s patches). (c) Multifocal form: groups of macrophages surrounded by lymphocytes in the lamina propria; the villous morphology is not strikingly altered. (d and e) Diffuse paucibacillary/lymphocytic form: marked thickening of the intestinal mucosa is due to the presence of a granulomatous infiltrate formed mainly by lymphocytes. Some scattered macrophages can be seen among them.

Histopathological characterization of the ileal lesions is shown in Figure-1. Of the 201 ilea, 36/201 (17.9%) were focal, 34/201 (16.9%) were multifocal, 18/201 (9.0%) were diffuse multibacillary/histiocytic, and 40/201 (19.9%) were diffuse paucibacillary/lymphocytic. The diffuse multibacillary/histiocytic form was detected only in AS sheep.

**Figure-2:**
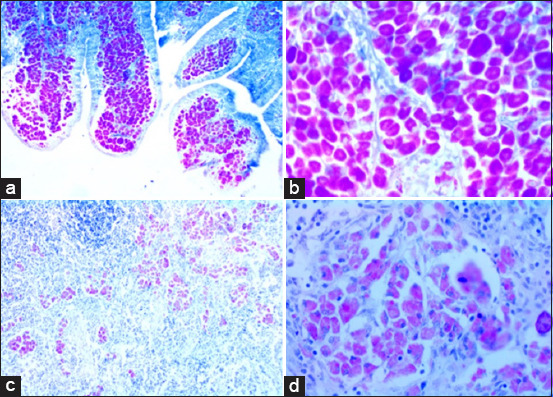
Ziehl–Neelsen staining of sheep intestinal tissue and mesenteric lymph nodes. (a) Acid–fast bacilli in epithelioid macrophages in the lamina propria of the ileum. (b) Mucosal infiltrate demonstrates large numbers of intracytoplasmic acid–fast bacilli in the ileum. (c) Acid–fast bacilli in epithelioid macrophages in the cortex of the mesenteric lymph node. (d) Granuloma containing epithelioid macrophages and large numbers of intracytoplasmic acid–fast bacilli in the mesenteric lymph node.

Histopathological examination of the ZN-stained sections of the ileum revealed acid–fast bacteria in different layers of the ileal tissue (Figure-2). The number and percentage of ileum samples with evidence of acid–fast bacteria were 104/201 (51.7%) for all sheep, 34/88 (38.6%) for TS sheep, and 70/113 (61.9%) for AS sheep.

**Figure-3:**
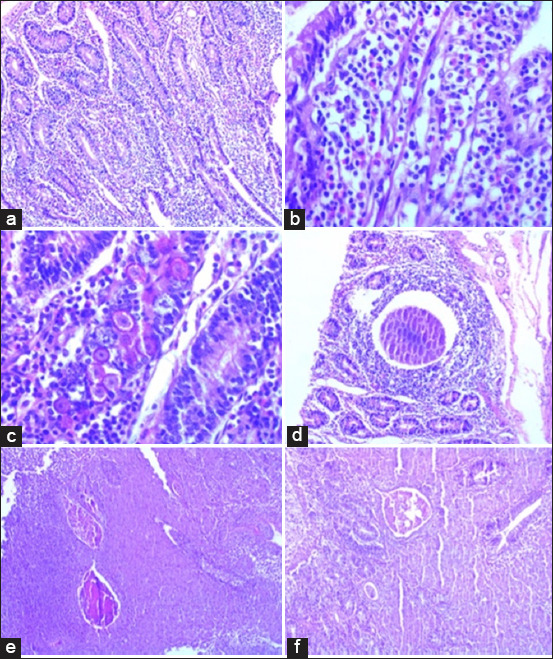
Histopathological examination of ileum sections stained with hematoxylin and eosin to detect eosinophilic inflammation and parasitic structures. (a and b) Severe eosinophilic inflammation (more than 20 eosinophils per field). (c) Eosinophils, lymphocytes, macrophages, and coccoidal gametocytes within the intestinal epithelium. (d) *Eimeria gilruthi* macromeronts in intestinal epithelium. (e and f) *Strongyle* spp. eggs in intestinal epithelium.

Histopathological examination of the ileum revealed variable degrees of eosinophilic inflammation and evidence of parasitic structures in both sheep breeds (Figure-3). Eosinophilic infiltration was detected in 178/201 (88.6%), 70/88 (79.5%), and 108/113 (95.6%) of all combined sheep, TS sheep, and AS sheep, respectively (Table-2). Severe eosino- philic inflammation was the most common score in both breeds. Parasitic structures were detected in 12/201 (6.0%), 2/88 (2.3%), and 10/113 (8.8%) of all combined sheep, TS sheep, and AS sheep, respectively (Table-3).

**Table-3:** Parasitic structures observed in hematoxylin and eosin stained ileum tissue samples.

Parasitic classification	Number (%)		

	All Sheep	Tsurcana sheep	Awassi sheep
None	189 (94)	86 (97.7)	103 (91.2)
Protozoa (*Eimeria* spp.)	4 (2.0)	1 (1.1)	3 (2.7)
Nematode (*Strongyle* and *Nematodirus* spp.)	7 (3.5)	1 (1.1)	6 (5.3)
Mixed infections (nematodes and protozoa)	1 (0.5)	0 (0)	1 (0.9)

**Figure-4:**
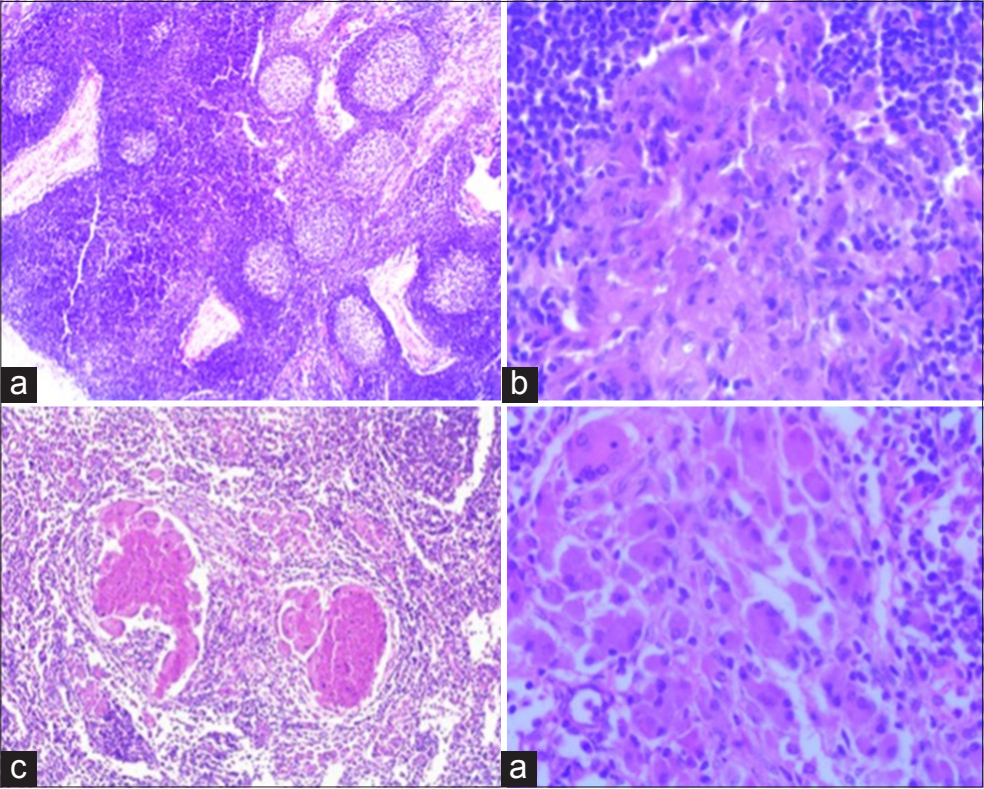
Histopathological lesions in the mesenteric lymph nodes caused by *Mycobacterium avium* subspecies *paratuberculosis* in sheep. (a) Severe follicular hyperplasia. (b) Granuloma in the paracortex contains a cluster of epithelioid cells. (c) Multifocal areas of caseous necrosis, clusters of epithelioid macrophages, and few giant cells near necrotic areas. (d) Cluster of epithelioid macrophages, giant cell, and lymphocytes.

Out of 192 mesenteric lymph nodes (all sheep combined), 136/192 (70.9%) had lesions compatible with Johne’s disease (Table-4). Histopathologically, lesions were characterized by epithelioid macrophage aggregation or clusters in the paracortical zone of the affected mesenteric lymph nodes (Figure-4). Microgranulomas were also seen in the paracortical zone and subscapular sinuses of mesenteric lymph nodes composed of epithelioid cells with or without giant cells. Focal to diffuse areas of caseous necrosis with calcification were also observed, and a few giant cells in the necrotic areas were detected in some samples. The number and percentage of samples with evidence of acid–fast bacteria in mesenteric lymph nodes were 137/192 (71.4%) for all sheep, 51/78 (65.4%) for TS sheep, and 86/114 (75.4%) for AS sheep.

**Table-4:** Johne’s disease histopathological lesions in the mesenteric lymph nodes.

Histopathological findings	Number (%)		

	All Sheep	Tsurcana sheep	Awassi sheep
None	56 (29.2)	27 (34.6)	29 (25.4)
Epitdelioid cells	124 (64.6)	48 (61.5)	76 (66.7)
Epitdelioid and giant cells	8 (4.2)	3 (3.8)	5 (4.4)
Epitdelioid cells, giant cells, and mineralization	4 (2.10)	0 (0)	4 (3.5)

### Fecal parasite examination

Positive fecal floatation results were recorded in 26/207 (12.6%) of all sheep breed samples; 11/89 (12.4%) of TS sheep, and 15/118 (12.7%) of AS sheep (Table-5).

**Table-5:** Fecal floatation parasitic examination.

Parasites	Number (%)		

	All sheep	Tsurcana sheep	Awassi sheep
None	181 (87.4)	78 (87.6)	103 (87.3)
Protozoa (*Eimeria* spp.)	11 (5.3)	10 (11.2)	1 (0.8)
Nematodes (*Strongyle* and *Nematodirus* spp.)	13 (6.3)	1 (1.1)	12 (10.2)
Mixed infections (nematodes and protozoa)	2 (1.0)	0 (0)	2 (1.7)

### Histological and fecal evidence of coinfection

The number and percentage of tissue sections with acid–fast bacteria and histological evidence of parasitic structures in the ileum were 10/201 (5.0%) of all sheep samples. The acid-fast bacteria and parasitic infection were recorded in 2/88 (2.3%) and 8/113 (7.1%) imported TS and local AS sheep, respectively.

The number and percentage of tissue sections with acid–fast bacteria and eosinophilic infiltration in the ileum were 99/104 (95.1%) in both sheep breeds, while they were detected together in 30/88 (34.1%) and 69/113 (61.6%) TS and AS sheep, respectively.

The number and percentage of tissue samples with acid–fast bacteria in the ileum and positive fecal floatation test were 17/201 (8.5%) of all sheep. The two conditions were recorded in 5/88 (5.7%) and 12/113 (10.6%) TS and AS sheep, respectively.

The number and percentage of tissue samples with acid–fast bacteria in the ileum and either positive histological evidence of parasitic structures or positive fecal floatation parasite tests were 26/201 (12.9%) of all sheep.

### Risk factor analysis

Multivariant regression analysis identified six potential risk factors for acid–fast bacteria in the ileum of both breeds. The presence of eosinophilic infiltration in the ileum, detection of histological evidence of parasitic structures in the ileum, positive fecal floatation test, detection of parasitic structures and positive fecal floatation test combined, and the detection of parasitic structures or positive fecal floatation test were associated with a statistically significant (p ≤ 0.05) increased risk for positive acid–fast bacteria in the ileum in both breeds. However, the final logistic regression model revealed that only the sheep breed was a statistically significant (p ≤ 0.05) risk factor for acid–fast bacteria in the ileum (Table-6). AS sheep were 2.164 times more likely to have acid–fast bacteria in ileal tissue samples. Furthermore, samples with eosinophilic inflammation were 2.874 times more likely to have acid–fast bacteria in the ileum.

**Table-6:** Multivariate logistic regression model of potential risk factors associated with the presence of acid–fast bacteria in the ileum (n = 201).

Risk factors	Positive (%)	OR	95% CI		p-value (χ^2^), **

			Lower	Upper	
Eosinophilic infiltration	99 (49.3)	2.874	0.984	8.394	0.054
Detection of parasitic structures	10 (5.0)	3.785	0.793	18.069	0.095
Positive fecal floatation test	17 (8.5)	2.388	0.917	6.218	0.075
The sheep breed	104 (51.7)	2.164	1.181	3.964	0.012

** p-value: Chi-square for the difference between the prevalence of AFB in the ileum or mesenteric lymph nodes and risk factors in small ruminants (statistically significant at p*≤*0.05 (two-sided)). Fisher’s exact test was performed instead of (χ^2^) when variables had expected count 5 in one or more cells. Chi-square test was not performed when zero number of samples was available. OR=Odds ratio, CI=Confidence interval

## Discussion

A previous study by Hailat *et al*. [14] has demonstrated that paratuberculosis is endemic in Jordan and causes tremendous financial losses, with a reported prevalence rate of 50%. A previous study by Hailat *et al*. [15] conducted in Jordan on paratuberculosis has focused mainly on comparing and improving various diagnostic laboratory techniques. In this study, gross examination revealed that larger numbers of local AS sheep had mucosal thickening of the ileum and enlargement and mineralization of the mesenteric lymph nodes compared with imported TS sheep. In this study, the infection rate of paratuberculosis in AS sheep was 9%. This rate has doubled during the last decade (4.81% [14]). These results could be attributed to an older population of AS sheep (the majority were older than 2 years of age compared to 1.5–2 years for the TS sheep) involved in this study compared to previous study populations [14]. The TS sheep breed used in this study was originally from Romania, and up to this point, there have been no studies regarding the diagnosis of paratuberculosis by histopathology in sheep from Romania. The prevalence of paratuberculosis in the Western area of Romania was 50.59% in cattle, 33.33% in goats, and 9.9% in sheep [16].

The severity of intestinal lesions was classified in this study based on the histological classification system of MAP lesions developed and used in bovine species. A similar classification system developed by Hailat *et al*. [14] consisted of four grades (I, II, III, and SP) based on different histological criteria, including the type and density of cellular infiltrates (lymphocytes, macrophages, and epithelioid cells) in the small intestine and mesenteric lymph nodes. Histopathological examination of intestinal lesions in this study revealed more severe lesions compatible with clinical paratuberculosis in AS sheep (71.7%) compared to TS sheep (53.4%). In addition, all diffuse multibacillary/histiocytic lesions were recorded in AS sheep (15.9%). These findings are because most samples from AS sheep were collected from older animals that had more time to develop the chronic disease [12].

Eosinophilic inflammation is an indicator of parasite infection [17]. The results of this study demonstrated that the prevalence of eosinophilic inflammation in H&E-stained tissues was extremely high (88.6%) in all sheep. In AS sheep, eosinophilic inflammation was recorded in 95.6% of the cases, which was higher than in TS sheep (79.5%). Both breeds exhibited severe eosinophilic inflammation in the ileum. This study found parasitic structures in H&E-stained ileum tissues more frequently in AS than in TS sheep. This can be attributed to the fact that most AS sheep involved in the study were older than those of the other breeds and, therefore, had a longer exposure time to develop parasitic infections. In addition, imported sheep breeds like TS sheep have been given more recent anthelmintic drugs to make them healthy for export to other countries [18]. The majority of parasites detected in this study were protozoan and nematode species. Previous studies by Tafti and Rashidi [19] and Pawar *et al*. [20] on goats indicated that the most common intestinal parasites were nematodes and cestodes, including *Haemonchus contortus, Haemonchus placei, Oesophagostomum columbianum, Trichuris ovis, Stilesia globipunctata, Avitellina centripunctata*, and *Moniezia expansa*.

In this study, most mesenteric lymph nodes exhibited histopathological changes consistent with lymphofollicular hyperplasia. AS sheep had more samples with histopathological lesions suggestive of paratuberculosis (74.6%) than TS sheep (65.4%). In addition, severe lymph node lesions, such as granulomatous formation and mineralization, were only observed in AS sheep. All histopathological findings are consistent with those of previous studies by Idris *et al*. [3] and Robbe-Austerman [21].

ZN findings in this study showed that 61.9% of ileum tissue sections from AS sheep were positive for acid–fast bacteria, compared with 38.6% in TS sheep. In both breeds, acid–fast bacteria were detected more frequently in mesenteric lymph nodes than in the ileum. These findings are inconsistent with previous studies by Hemalatha *et al*. [22] and Sikandar *et al*. [23], in which intestinal tissue sections repor- tedly had more positive ZN results than mesenteric lymph nodes. Acid–fast bacteria were observed inside the cytoplasm of macrophages and in epithelioid cells or free outside the cells. In previous studies by Hailat *et al*. [14, 15] in Jordan, ZN staining of intestinal tissue staining has shown that only 11% of tissue samples were positive in ZN staining. Only samples traced back to grades II, III, and SP were positive [14]. Other studies by Idris *et al*. [3] and Velez-Hoyos and Jimenez-Tobon [24] have shown that ZN staining has low specificity and sensitivity because of difficulties in differentiating MAP from other acid–fast bacteria (*Mycobacteria* spp., *Nocardia* spp., etc.). Acid–fast bacteria were detected by ZN staining in only 4 of 12 ELISA-positive small ruminants [25]. Nonetheless, ZN staining is considered an easy, fast, and economical diagnostic method and can be employed for the initial screening of MAP [3].

The fecal floatation test in this study was used to screen for both nematode eggs and coccidian oocysts, as this technique is known to be the most common concentration technique with good sensitivity [26]. This result revealed that protozoal infection with *Eimeria* spp. was more frequent in TS sheep, and only one TS sheep sample had *Strongyle* spp. eggs. This is attributed to the fact that imported sheep are routinely dewormed before transport [16]. Furthermore, TS sheep suffer periods of stress during shipping to Jordan, which makes them prone to the shedding of *Eimeria* spp. oocysts [27]. In addition, *Eimeria* spp. is the most prevalent parasitic infection in all age categories of goats in Romania [28].

In this study, nematode infection was observed more frequently in AS sheep; this could be a result of the timing of sample collection, which occurred during spring and summer. Sheep are typically more exposed and vulnerable to nematode parasitic infections during this period [29]. Furthermore, AS sheep showed more positive parasitological evidence, either by H&E-stained tissue sections or fecal floatation. A study by Al-Qudah *et al*. [30] on goats in Jordan revealed that gastrointestinal parasitism was detected in 45% of slaughtered goats. The observed parasites were *Trichostrongylus, Haemonchosis, Dictyocaulus filaria, Echinococcosis*, and *Eimeria* spp. [30]. Another study by Abo-Shehada and Abo-Farieha [31] conducted in Jordan demonstrated that the prevalence of *Eimeria* spp. in goats was 54%. These results are in congruence with the results reported here for AS sheep.

Acid–fast bacteria in tissue sections from the ileum and eosinophilic inflammation of the intestine were detected more frequently in AS sheep (61.6%) compared to TS sheep (34.1%). These findings could be attributed to samples collected from older AS sheep compared to TS sheep. Collectively, 95.1% of sheep samples from both breeds exhibited acid–fast bacteria on ZN staining and eosinophilic inflammation. At the same time, 98.6% of AS sheep ileum samples that showed positive acid–fast bacteria on ZN staining had eosinophilic inflammation, which was higher than that in TS sheep (88.2%). Eosinophil infiltrates have been observed in some models of paratuberculosis [32–34]. Furthermore, samples positive for acid–fast bacteria in the ileum and fecal floatation were detected more frequently in AS sheep than in TS sheep. The aforementioned results could be attributed to the AS sheep breed being more predisposed to paratuberculosis and parasitic infections. Previously, it was hypothesized that eosinophils may enhance MAP infection by inhibiting macrophage activation and providing a suitable microenvironment for mycobacterial growth [34, 35]. However, more research is required into the relationship between intestinal parasitism and paratuberculosis to understand this interaction fully.

The multivariate logistic regression analysis in this study revealed six potential risk factors (p ≤ 0.05) for the outcome of acid–fast bacteria in ileum tissue sections in both sheep breeds. However, the final logistic regression model of all sheep breeds combined revealed that only the sheep breed was a statistically significant (p ≤ 0.05) risk factor. AS sheep were 2.164 times more likely to have acid–fast bacteria in ileal tissue sections. This finding was also evidenced by gross, histopathological (H&E), and ZN-stained tissue sections.

Furthermore, samples with eosinophilic inflammation were 2.874 times more likely to have acid–fast bacteria in the ileum. Previously, only two studies by Mir *et al*. [36] and Naranjo *et al*. [37] explored the coinfection relationship between paratuberculosis and parasites. In an experimental study by Naranjo-Lucena *et al*. [38], animals co-infected with *Fasciola hepatica*, and MAP showed reduced peripheral mononuclear cell proliferation and a significant reduction in ileocecal lymph node leukocyte proliferation in response to MAP antigens. It was reported that *F. hepatica* products reduced the expression of CD14 receptors by macrophages and increased apoptosis and bacterial (MAP) uptake [38]. In another naturally occurring outbreak of concurrent infection among goats with visceral linguatulosis and paratuberculosis, it was concluded that infection with *Linguatula serrata* predisposed them to a multibacillary form of paratuberculosis [36].

This is the first study conducted in Jordan regarding parasite coinfection with paratuberculosis in naturally infected sheep. Furthermore, it discussed the multiple risk factors associated with paratuberculosis. Collecting samples from older animals was the main limitation of this study since the disease is chronic.

## Conclusion

The results of this preliminary study indicate that coinfection between gastrointestinal parasites and paratuberculosis may be associated with increased shedding and transmission of paratuberculosis in natural settings. It was also shown that the sheep breed plays a critical role in the detection of MAP in ZN-stained ileum tissue sections. The AS breed of sheep had an increased risk of contracting paratuberculosis compared with the TS breed. This study is unique, as it is one of the few in the world to explore the dynamics between parasites, eosinophilic inflammation, and paratuberculosis in naturally infected sheep and their implications for disease pathology.

## Authors’ Contributions

RMM and WMH: Planned and designed the experiments and drafted and revised the manuscript. AR: Data analysis and fieldwork. ZBI: Analyzed the data and drafted, reviewed, and edited the manuscript. All authors have read and approved the final manuscript.
